# Integrating Multi-Task Eye Tracking and Interpretable Machine Learning for High-Accuracy Screening of Amblyopia in Pediatric Populations

**DOI:** 10.3390/jemr19020026

**Published:** 2026-03-02

**Authors:** Xiumei Song, Yunhan Zhang, Hongyu Chen, Chenyu Tang, Bohan Yao, Hubin Zhao, Luigi G. Occhipinti, Arokia Nathan, Changbin Zhai, Shuo Gao

**Affiliations:** 1 Beijing Tongren Eye Center, Beijing Tongren Hospital, Beijing Ophthalmology & Visual Science Key Laboratory, Capital Medical University, Beijing 100730, China; songxm123@yeah.net (X.S.); rfchenhongyu2006@163.com (H.C.); 2 School of Instrumentation and Optoelectronics Engineering, Beihang University, Beijing 100191, China; 24373304@buaa.edu.cn; 3 Department of Engineering, University of Cambridge, Cambridge CB3 0FA, UK; ct631@cam.ac.uk (C.T.); lgo23@cam.ac.uk (L.G.O.); 4 West China Medical Center, Sichuan University, Chengdu 610065, China; hk1911905442@163.com; 5 Faculty of Medical Sciences, University College London, London WC1E 6DD, UK; hubin.zhao@ucl.ac.uk; 6 Darwin College, University of Cambridge, Cambridge CB3 9EU, UK; an299@eng.cam.ac.uk

**Keywords:** amblyopia, eye tracking, oculomotor behavior, pediatric screening, random forest classification, smooth pursuit

## Abstract

Amblyopia is a developmental disorder of spatial vision in which abnormal visual experience leads to persistent reductions in acuity and contrast sensitivity, even after optimal optical correction. We introduce a brief, child-friendly battery of task-evoked eye tracking that probes fixation stability, fine pattern processing, and smooth pursuit control across three simple paradigms. Oculomotor traces are transformed into physiologically interpretable markers—fixation dispersion and saccadic strategy, orientation-dependent drift and stability, pursuit gain, and tracking error—and used to train a compact classifier with subject-wise validation and probability calibration. In a cohort of school-aged participants with clinically diagnosed unilateral amblyopia and age-matched visually normal controls tested under best-corrected viewing conditions, the approach consistently separated groups with stable performance across folds; feature-importance analyses indicated that pursuit- and orientation-dependent markers contributed most. The protocol runs in minutes, is objective and noninvasive, and is well tolerated in pediatric settings. By quantifying functional consequences of amblyopic vision that complement conventional acuity testing, this work positions task-evoked eye movements as practical biomarkers for screening and monitoring, and lays the groundwork for prospective validation and age-stratified norms in community and school-based vision care.

## 1. Introduction

Amblyopia is a common developmental visual disorder characterized by reduced visual function that cannot be fully explained by structural abnormalities of the eye and is typically associated with abnormal visual experience during early childhood (e.g., anisometropia, strabismus, or both) [1,2,3]. Because neural plasticity is highest in early life, timely detection and intervention are critical: earlier treatment is associated with better outcomes, whereas delayed diagnosis increases the risk of persistent visual impairment and reduced binocular function into adulthood [4,5]. Despite this, population-level screening remains challenging. Conventional screening often relies on monocular visual acuity testing, stereoacuity, and/or instrument-based photo screening, each with practical limitations in young children, variable sensitivity across amblyopia etiologies, and dependence on attention, cooperation, and trained personnel [6,7]. In busy clinics or community settings, there is a pressing need for child-friendly, time-efficient, and objective screening tools that can be deployed with minimal burden while still capturing functional markers relevant to amblyopia [8].

Beyond reduced acuity, amblyopia is associated with characteristic alterations in oculomotor behavior [9]. A growing body of work suggests that fixation stability, microscale drift, saccadic control, and smooth-pursuit fidelity can be compromised, reflecting altered sensory encoding and visuomotor integration [10,11]. Importantly, these oculomotor signatures can persist even under binocular viewing because binocular fusion and the dominant (fellow) eye do not necessarily normalize the amblyopic eye’s sensory noise, interocular imbalance, or motor control adaptations acquired during development [12,13,14,15]. Such findings motivate oculomotor assessment as an attractive complement to acuity-based screening: eye movements provide a continuous, quantitative readout of the visual system during naturalistic viewing and can be elicited by short tasks that are engaging for children [16,17,18,19,20,21].

Eye tracking provides a practical measurement modality for such oculomotor biomarkers [22,23,24]. Early studies using laboratory-grade trackers under head restraint demonstrated that amblyopia can be associated with increased fixation instability, abnormal saccadic latencies, altered pursuit gain, and more frequent catch-up saccades [25,26,27,28,29]. However, translating these insights to screening faces several barriers. First, many laboratory protocols use specialized equipment, high sampling rates, and rigid head stabilization, which are not easily compatible with child screening in natural settings. Second, tasks that isolate a single oculomotor component may not be sufficiently robust in real-world deployment because children differ in compliance, fatigue, and attentional state [30,31,32,33]. Third, algorithmic pipelines can be sensitive to sampling rate and preprocessing choices; metrics such as microsaccade rate and fine-grained velocity profiles are difficult to estimate reliably at low sampling rates typical of lightweight wearable devices [34,35,36]. Finally, reported model performance in small cohorts can be inflated by methodological pitfalls, particularly information leakage when trial-level samples from the same subject are split across training and testing, or when preprocessing and operating-point selection implicitly use held-out subjects. For screening applications, rigorous subject-wise evaluation and transparent reporting are therefore essential [37].

An additional practical question concerns how to leverage binocular eye tracking for unilateral amblyopia. In screening contexts, children typically view stimuli binocularly (both eyes open) under habitual correction. This condition is ecologically valid, but it raises a methodological choice: whether to model the amblyopic-eye stream, the fellow-eye stream, or a binocularly combined (“cyclopean”) gaze estimate. Cyclopean representations can improve robustness to transient tracking loss and reflect integrated visuomotor output during natural binocular viewing; however, they may also attenuate amblyopia signatures if the fellow eye dominates control [24]. Addressing this trade-off requires clear signal definition, principled missing-data handling, and sensitivity analyses that compare performance across gaze-stream choices.

Motivated by these considerations, we propose a child-oriented screening framework that combines a lightweight wearable binocular eye tracker, a brief task-evoked protocol, and an interpretable machine-learning model evaluated under a strict subject-wise design. The protocol comprises three short tasks that probe complementary oculomotor behaviors relevant to amblyopia: (1) visual enumeration/search to capture scanning and stability, (2) orientation-dependent grating viewing to probe gaze stabilization, and (3) two-dimensional smooth pursuit to quantify eye–target coordination and corrective saccades. Together, the tasks yield clinically interpretable features that summarize gaze stability, saccadic organization, and pursuit fidelity, improving robustness beyond any single task.

**Figure 1:**
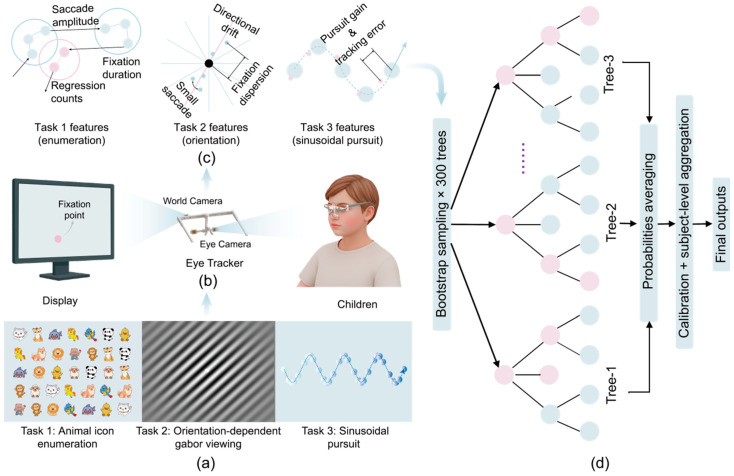
Schematic overview of the proposed screening pipeline. (**a**) Children perform three visual tasks. (**b**) Binocular gaze signals are recorded using a wearable eye tracker. (**c**) Extraction of task-specific oculomotor features. (**d**) A calibrated Random Forest classifier.

To obtain reproducible estimates, we use a nested, strictly subject-wise evaluation pipeline (see Figure 1). Standardization, imputation, model fitting, probability calibration, and operating-point selection are learned from training subjects only within each split. We use a Random Forest as the primary classifier and calibrate probabilities with isotonic regression. We report discrimination (AUC, average precision) and operating-point metrics (sensitivity, specificity, and accuracy) with uncertainty estimates, and translate sensitivity/specificity into expected PPV/NPV across plausible prevalence values.

We evaluate this framework in school-aged children with unilateral amblyopia and age-matched controls under best-corrected binocular viewing. Our contributions are threefold: (1) a practical wearable binocular eye-tracking protocol suitable for children; (2) an interpretable multi-task feature representation evaluated with leakage-resistant nested subject-wise validation and calibrated thresholding; and (3) screening-oriented analyses including baseline comparisons, task ablation/feature importance, robustness to preprocessing choices, and sensitivity to gaze-stream definition (cyclopean vs. eye-specific). These results support task-evoked wearable eye tracking as a quantitative screening approach for unilateral amblyopia.

### 1.1. Hardware Description

We employed a custom wearable binocular eye-tracking prototype optimized for school-aged children and natural head posture during short screening-style tasks. The device integrates three identical off-the-shelf USB camera modules in a lightweight head-mounted frame: two inward-facing cameras (one per eye) for infrared (IR) eye imaging and pupil localization, and one outward-facing world camera to capture the stimulus display for screen localization.

All three cameras used the same module and optics (12-megapixel sensor; maximum resolution 3840 × 3104 pixels; 3.95 mm lens; approximately 80° field of view). For eye imaging, the inward-facing cameras operated with integrated 940 nm IR illumination to provide stable pupil observation while reducing sensitivity to ambient visible-light variation.

The gaze signal for subsequent analyses was produced at 30 Hz. This sampling configuration preserves high eye-image resolution for reliable pupil-center localization while maintaining a compact and lightweight wearable design. All downstream analyses were designed to rely on oculomotor descriptors that are robust at 30 Hz (e.g., gross fixation dispersion, scan-path statistics, and pursuit fidelity), rather than fine-scale microsaccadic dynamics.

A Python 3.9-based host application performed synchronized acquisition and online processing. At each time step, pupil centers were detected independently in the left- and right-eye IR image streams and mapped to gaze locations in display coordinates using per-eye calibration functions. To maintain a stable definition of screen coordinates under natural head motion, ArUco fiducial markers were placed on and around the display and detected in the world-camera stream. Marker corners were used to estimate the screen plane and compute a planar transformation between world-camera pixels and the known screen coordinate system. Because the world camera was rigidly mounted to the eye cameras, updating the screen plane in the world-camera frame provides a consistent gaze-to-screen mapping under minor pose variations.

Visual stimuli were presented on a 24-inch liquid-crystal display (1920 × 1080 pixels; 60 Hz) at a viewing distance of 60 cm. The monitor was luminance-calibrated before testing (white field 120 cd/m^2^; gamma 2.2) to ensure repeatable photometric conditions across participants and sessions.

Calibration used a child-friendly five-point dynamic procedure. Five screen locations were displayed sequentially (800 ms per location). For each eye, we fitted a second-order two-dimensional polynomial mapping from pupil-center coordinates in eye-image space to gaze locations in screen coordinates using least-squares regression. The calibration sequence could be repeated once when necessary. Calibration was accepted when the mean screen-plane error did not exceed 0.8° of visual angle.

Rigid head restraint (e.g., chin rest) was not imposed. Participants were instructed to maintain an upright posture and to keep their head as still as possible. Calibration was repeated if noticeable drift occurred. Analyses were restricted to trials in which gaze remained within the calibrated region.

### 1.2. Participants and Clinical Assessment

Seventy school-aged participants were recruited from the ophthalmology outpatient clinic and affiliated community health centers of Beijing Tongren Hospital, including 35 children with clinically diagnosed unilateral amblyopia and 35 age-matched visually normal controls. Demographic and clinical characteristics are summarized in Table 1.

All participants underwent a comprehensive clinical assessment performed by a pediatric ophthalmologist, including best-corrected visual acuity (BCVA), refraction, ocular alignment evaluation, and stereopsis testing, following routine clinical protocols at our center. BCVA was recorded for each eye under habitual optical correction and reported in logMAR units. Amblyopia etiology was classified as anisometropic, strabismic, or mixed based on clinical findings (Table 1).

Amblyopia-group inclusion criteria were: (i) unilateral amblyopia diagnosed clinically; (ii) a BCVA in the amblyopic eye between 0.2 and 0.5 logMAR (approximately 0.3–0.6 decimal) with an interocular acuity difference of at least 0.2 logMAR; and (iii) anisometropia, strabismus, or a mixed mechanism identified as the underlying cause. Exclusion criteria included a history of intraocular surgery, ocular pathology other than amblyopia, or neurological disease.

Control-group inclusion criteria were a BCVA of at least 0.8 (decimal; ≤0.1 logMAR) in each eye with an interocular difference of at most 0.1 logMAR, normal binocular alignment and stereopsis, and emmetropia or minimal refractive error.

All eye-tracking tasks were performed binocularly with both eyes open while children wore their habitual optical correction (spectacles or contact lenses, when prescribed) that achieved their enrollment BCVA, so that the recordings reflected best-corrected function rather than uncorrected blur. Children with known cognitive impairment or a history of neurological or psychiatric disorders were excluded. The study protocol was approved by the Institutional Ethics Committee of Beijing Tongren Hospital, and written informed consent was obtained from parents/guardians in accordance with the Declaration of Helsinki.

### 1.3. Experimental Paradigm

To probe oculomotor impairments associated with unilateral amblyopia under binocular viewing conditions, we designed a three-stage, task-driven protocol comprising (1) an animal icon enumeration task, (2) an orientation-dependent grating viewing task, and (3) a dynamic sinusoidal pursuit task. All tasks were presented on the calibrated LCD display in a quiet, dimly lit room at a viewing distance of 60 cm. Eye movements were recorded binocularly and continuously throughout the experiment.

Each task was organized into discrete trials defined by task-specific onset and offset events. Practice trials were provided before each task to ensure that children understood the instructions and response requirements. Short breaks (~30 s) were provided between tasks, and additional brief pauses were allowed as needed to maintain attention and comfort. The task structure and timing are shown in Table 2.

#### 1.3.1. Task 1: Animal Icon Enumeration

**Figure 2:**
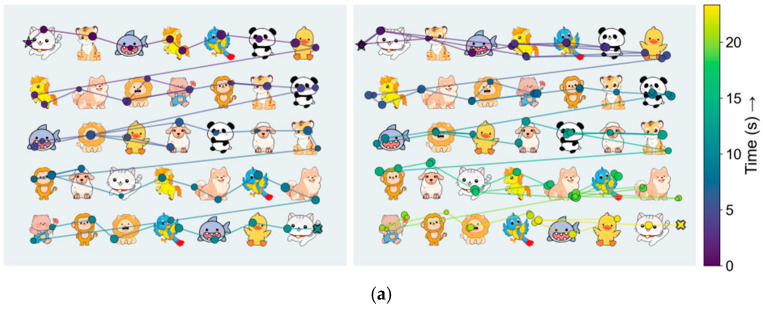

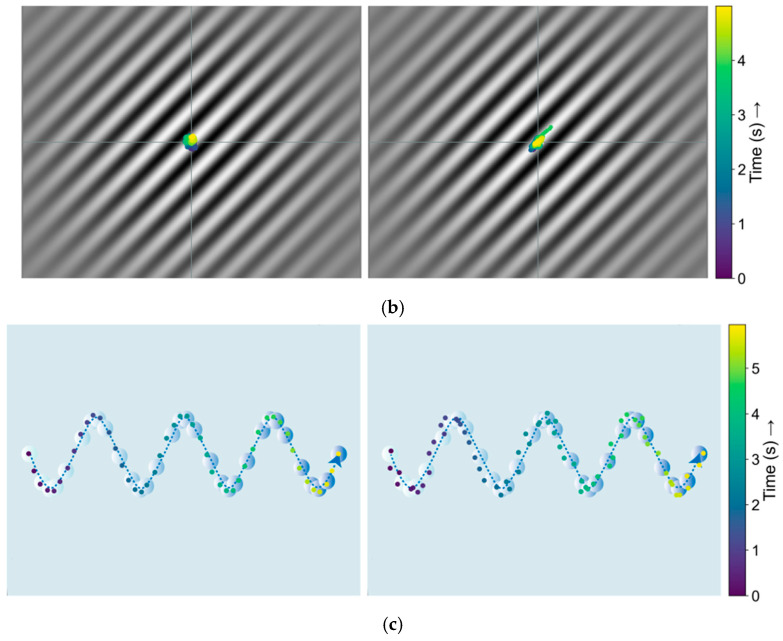
Task-evoked visual stimuli and representative gaze trajectories for the three oculomotor tasks. Each row shows binocular gaze data from a visually normal control child (**left**) and an amblyopic child (**right**). (**a**) Animal icon enumeration arrays, where children freely scan the display to count the cued animal category; colored traces indicate the time course of gaze during one trial. (**b**) Static oriented grating presented at the center of the screen; dots show successive gaze samples during attempted fixation. (**c**) Two-dimensional sinusoidal pursuit of a moving target; dots indicate gaze position over time as the child tracks the target. In all panels, color encodes time from trial onset (see color bars), and traces are shown after removal of blinks and off-screen samples.

In Task 1 (see Figure 2a), participants viewed a child-friendly array containing multiple cartoon animal categories arranged with uniform spacing on a neutral background. At trial onset, an on-screen prompt specified the target category (e.g., “panda”). Participants freely explored the array to count the number of target animals and reported the total via keypress/button response. The array remained on screen until the response or a maximum of 60 s.

This task was designed to quantify fixation stability, search efficiency, and saccadic strategy (including regressions and refixations) under naturalistic exploration. Because the array contains fine spatial features (edges and textures), the task imposes demands on spatial vision and attention that may elicit compensatory search patterns in children with amblyopia even during best-corrected binocular viewing [38,39].

#### 1.3.2. Task 2: Orientation-Dependent Grating Viewing

Task 2 (see Figure 2b) assessed orientation-dependent pattern processing using centrally presented, static Gabor patches (Gaussian-windowed sinusoidal gratings). Each patch subtended 3° of visual angle and had a spatial frequency of 6 cycles/degree. Stimuli were presented at 12 orientations (0°, 15°, 30°, …, 165°) and at three suprathreshold Michelson contrast levels (0.2, 0.4, and 0.8), in pseudorandom order.

In each trial, the Gabor stimulus was displayed for 2 s against a mean-luminance background, followed by a 1 s blank interval. The carrier phase was randomized at each presentation to discourage reliance on local luminance cues. To ensure engagement, participants provided a coarse orientation report using four arrow keys mapped to four orientation bins (horizontal, vertical, left-oblique, and right-oblique). Behavioral responses were recorded to confirm task compliance but were not used as predictors in the present analysis.

The formal Task 2 block comprised 72 trials (12 orientations × 3 contrasts × 2 repetitions), presented in pseudorandom order. The primary analyses focus on orientation-dependent oculomotor behavior (e.g., fixation stability and drift) during stimulus viewing rather than psychophysical threshold estimation [40].

#### 1.3.3. Task 3: Smooth Pursuit of a Sinusoidal Trajectory

In Task 3 (see Figure 2c), participants tracked a circular target (diameter 0.5°) moving along a two-dimensional sinusoidal trajectory for 20 s. The horizontal component had a peak-to-peak amplitude of approximately 30° (±15° from screen center), and the vertical component had a peak-to-peak amplitude of approximately 15° (±7.5° from center), yielding a maximum tangential velocity of approximately 10°/s within the central visual field. Random phase offsets were used across trials to reduce purely predictive tracking. A central fixation cross was presented for 3 s before each trial.

This task evaluated pursuit fidelity and eye–target coordination under binocular viewing. We expected larger tracking error, reduced pursuit gain, and more frequent corrective (catch-up) saccades in children with amblyopia due to reduced fidelity of motion processing and oculomotor control when one eye provides degraded input.

### 1.4. Task Sequence and Balance

The three tasks were administered in a fixed order (Task 1 → Task 2 → Task 3) to standardize the screening procedure and reduce instruction switching for children. The sequence progressed from a self-paced visual search task to brief discrete fixation trials and then to continuous smooth pursuit, allowing participants to become familiar with the display, response mapping, and head-mounted tracker before the continuous tracking block. Short inter-task breaks (~30 s) were provided, and additional breaks were allowed as needed.

To address potential fatigue or learning effects associated with the fixed order, trial order and timing were logged. Data-quality indicators were monitored throughout the session, including the proportion of invalid samples per trial (off-screen samples and samples removed due to blinks or tracking loss) and trial exclusion rates under predefined quality criteria. Order effects were evaluated by comparing early versus late trials within each task. These analyses are described in Section 1.6 and reported in the results.

All tasks were implemented in PsychoPy (v2023.2) and presented at a resolution of 1920 × 1080 pixels with a 60 Hz refresh rate. Stimulus timing was validated prior to data collection using a photodiode to confirm frame-locked onsets and accurate stimulus durations.

### 1.5. Signal Processing and Algorithm Design

The eye-tracking dataset provides time-resolved oculomotor information that can be summarized into clinically interpretable descriptors of gaze stability, saccadic strategy, and pursuit fidelity. We designed an analysis pipeline that transforms raw binocular gaze streams into fixed-length trial-level feature vectors and integrates these features into supervised classifiers to distinguish children with unilateral amblyopia from visually normal controls.

To ensure that reported performance reflects generalization to unseen subjects, all preprocessing transformations, model fitting, probability calibration, and operating-point selection were performed under a strictly subject-wise evaluation scheme. The following subsections describe gaze-signal definition and preprocessing, task-specific feature extraction, and the model development and evaluation protocol.

#### 1.5.1. Gaze Signal Definition and Preprocessing

The wearable tracker outputs two-dimensional gaze positions for the left and right eyes in screen coordinates at 30 Hz. Gaze coordinates were converted from pixels to degrees of visual angle using the known screen geometry and viewing distance so that downstream features were computed in angular units and were comparable across participants.

Primary analyses used a cyclopean gaze signal to reflect binocular viewing during screening. When both eyes provided valid samples at a given timestamp, cyclopean gaze was computed as the sample-wise mean of the two eye positions. When only one eye sample was valid, that eye’s gaze sample was used. When neither eye was valid, the sample was treated as missing. In secondary analyses, features were recomputed using the amblyopic-eye stream and fellow-eye stream separately (based on clinical labeling) to assess dependence on the gaze definition.

Invalid samples were identified using device validity indicators (e.g., pupil-loss flags) and a kinematic plausibility rule. Samples were marked invalid if associated with tracking loss, if they fell outside the display region, or if the instantaneous angular velocity exceeded 1000°/s (indicative of transient tracking glitches). To reduce blink-edge artifacts, samples within ±1 frame (±33 ms) of invalid segments were also excluded. The cleaned gaze stream was segmented into trials according to task-defined boundaries.

Missing-data handling was designed to preserve robustness at 30 Hz without introducing artificial smoothness. Gaps of up to 200 ms (≤6 consecutive missing samples) were bridged by linear interpolation; longer gaps were retained as missing. Trials were excluded if more than 30% of samples were invalid or missing after cleaning. These criteria ensure that dispersion- and event-related measures are computed from sufficiently continuous data.

To suppress high-frequency jitter while retaining task-relevant trends, gaze position was smoothed using a Savitzky–Golay filter (window length: five samples; polynomial order 2). Velocity was then estimated by a central finite difference on the smoothed position signals. For the pursuit task, target velocity was computed analytically from the known trajectory and sampling times. All event-related metrics were interpreted at a coarse temporal resolution consistent with 30 Hz sampling.

More detailed parameters are provided in Table 3. 

#### 1.5.2. Feature Extraction

Raw gaze samples were recorded as two-dimensional coordinate sequences (x, y) and segmented into trials. For each trial, we computed a fixed-length feature vector summarizing gaze stability, saccadic strategy, and pursuit fidelity. Unless otherwise specified, angular quantities are expressed in degrees of visual angle.

Task 1 (visual enumeration) features characterized free-viewing behavior. Fixations were segmented using an I-DT algorithm (dispersion threshold 1.0°; minimum duration 100 ms). Fixation-duration statistics (mean and standard deviation), fixation dispersion (within-fixation RMS), and the number of refixations were computed per trial. Saccade amplitude was defined as the Euclidean distance between successive fixation centroids. Regression counts were computed as the number of saccades directed opposite to the subject’s predominant horizontal scanning direction within the array. Scan-path entropy quantified the unpredictability of the fixation-to-fixation transition sequence: fixation centroids were mapped to a uniform 8 × 6 grid over the display, and Shannon entropy was computed over the resulting state-transition sequence.

Task 2 (grating viewing) features characterized fixation stability and orientation-dependent drift during stimulus viewing. Trials were analyzed within a circular region of interest (ROI) centered on the stimulus (radius 2.0°). Fixation latency was defined as the time from stimulus onset to the first fixation whose centroid fell inside the ROI. Directional drift was computed as the net gaze displacement projected onto the stimulus orientation axis within the trial. Fixation dispersion was quantified as the area of the convex hull of gaze samples within the ROI, providing a robust measure of spatial spread over the 2 s viewing period.

To quantify small corrective behavior at a resolution compatible with 30 Hz, we defined small corrective saccade-like events using velocity thresholding on the smoothed gaze signal. A saccade-like event was detected when instantaneous speed exceeded 60°/s for at least one sample; event amplitude was defined as the position change between the samples immediately before and after the threshold crossing. Events with an amplitude between 0.3° and 1.0° were counted as small corrective saccade-like events. The resulting count was normalized by trial duration and reported as a rate (min^−1^). This metric is interpreted as a coarse index of small corrective behavior rather than a precise estimate of canonical microsaccades.

Task 3 (sinusoidal pursuit) features were computed by aligning gaze samples to the known target trajectory frame by frame. Tracking error was computed as the mean Euclidean distance between gaze position and target center over the trial. Pursuit gain was computed as the median ratio of gaze speed to target speed for samples where the target speed exceeded 2°/s, reducing instability near velocity reversals. Catch-up saccades were defined as saccade-like events with an amplitude greater than 1.5° that reduced instantaneous position error. Pursuit onset latency was defined as the first time after motion onset at which the gaze velocity exceeded 5°/s and remained directionally consistent with the target for at least three consecutive samples.

Trial-level feature vectors extracted from the three tasks were concatenated into a single representation for classification. Feature dimensionality was kept modest (dozens of features) to match the cohort size and to facilitate interpretability.

#### 1.5.3. Model Development and Evaluation

Trial-level feature vectors extracted from the three tasks were concatenated into a single representation, $x_{i,t}\in R^{p}$, where i indexes subjects and t indexes trials. Within each cross-validation split, features were standardized by z-scoring using normalization parameters estimated only from training subjects and then applied unchanged to validation/test subjects. Missing feature values (e.g., due to trial exclusion after quality control) were imputed using feature-wise medians learned only from the training data, with the learned mapping applied consistently to held-out subjects to avoid information leakage.

The primary classifier was a Random Forest composed of M = 300 decision trees trained on bootstrap-resampled trials from the training subjects. Splits were selected by minimizing Gini impurity, and candidate features were randomly subsampled at each node ($max\_feature\;=\;\sqrt{p}$) to encourage diversity among trees. Class weights were set inversely proportional to class frequencies within each training set. For subject i and trial t, the forest produced a trial-level posterior probability by averaging the tree-level posteriors:

(1) $$p_{i,t}=\frac{1}{M}\sum_{m=1}^{M}h_{m}\left(x_{i,t}\right),$$

where $h_{m}\left(\cdot \right)$ denotes the probabilistic output of the *m*-th tree.

Because Random Forest probabilities may be miscalibrated, trial-level probabilities were calibrated using isotonic regression learned within the training data. Denoting the calibration map by $g\left(\cdot \right)$, the calibrated trial probability is

(2) $${\tilde{p}}_{i,t}=g\left(p_{i,t}\right).$$

Importantly, $g\left(\cdot \right)$ was fitted exclusively in the inner cross-validation loop (described below) using only training subjects and then applied unchanged to the held-out subject in the outer test fold.

Subject-level evidence was obtained by robustly aggregating calibrated trial probabilities for each subject. The primary analysis used the median across all valid trials available for that subject:

(3) $${\tilde{p}}_{i}=median_{t}\left({\tilde{p}}_{i,t}\right),$$

A binary decision for subject i was then produced by thresholding the aggregated probability:

(4) $${\hat{y}}_{i}=I\left\{{\tilde{p}}_{i}\geq \tau \right\}.$$

where $I\left(\cdot \right)$ is the indicator function and τ is the operating threshold. The threshold τ was selected exclusively within the inner training data by maximizing Youden’s J statistic (J = sensitivity + specificity − 1) and was then applied unchanged to the held-out subject in the outer test fold.

Model selection and performance evaluation followed a nested, subject-wise protocol to ensure that all learning steps were confined to training subjects. Specifically, an outer leave-one-subject-out loop was used for testing, and within each outer training set, inner group-wise cross-validation over the remaining subjects was used to tune hyperparameters, fit the isotonic calibration map, and select the operating threshold. All preprocessing steps (standardization and imputation), calibration, and threshold selection were performed strictly within the training folds and then transferred to the corresponding held-out data. Performance was reported at the subject level using discrimination metrics (area under the ROC curve, AUC; and average precision, AP) and operating-point metrics (sensitivity, specificity, and accuracy). Confidence intervals for sensitivity and specificity were computed using binomial intervals (Wilson method), and confidence intervals for AUC/AP were computed by subject-level bootstrap resampling to respect the grouped structure of the data.

To contextualize the proposed model, baseline classifiers were trained on the same gaze feature set under the same nested, subject-wise evaluation protocol, including regularized logistic regression and a linear support vector machine; additional non-linear baselines (e.g., gradient-boosted decision trees) were evaluated using the same preprocessing, calibration, and threshold-selection procedures to enable fair comparison. In addition, to quantify the incremental value of gaze dynamics beyond routine clinical measures, we constructed (i) a clinical-only baseline and (ii) a combined clinical + gaze model using the same subject-wise evaluation protocol. The clinical feature set included best-corrected visual acuity (logMAR) in each eye, interocular acuity difference (IOD, logMAR), stereoacuity (arcsec; log-transformed), and refractive error summarized as spherical equivalent (SE, diopters) for each eye and anisometropia ($\left|{SE}_{R}-\;{SE}_{L}\right|$). For the clinical-only baseline, a regularized logistic regression classifier was trained on subject-level clinical vectors. For the combined model, clinical features were concatenated with subject-level aggregated gaze features (median across retained trials per task, then concatenated across tasks), with all preprocessing and model selection performed within the training folds only.

### 1.6. Statistical Analysis

Descriptive group comparisons of demographic and clinical variables followed Table 1: independent-samples *t*-tests were used for approximately normally distributed continuous variables, Mann–Whitney U tests for non-normal variables (stereoacuity), and chi-square tests for categorical variables (sex).

For feature-level group comparisons (when reported), trial-level features were first aggregated to the subject level (median across valid trials within each task; Task 2 features were additionally summarized across orientations). Between-group differences were assessed using independent-samples *t*-tests or Mann–Whitney U tests depending on normality (Shapiro–Wilk test). Effect sizes were summarized using Cohen’s d (*t*-tests) or rank-biserial correlation (Mann–Whitney U). When multiple features were tested, the false-discovery rate (FDR) was controlled using the Benjamini–Hochberg procedure (q = 0.05).

Order effects associated with the fixed task sequence were assessed using data quality and oculomotor summaries. Within each task, we compared early versus late trials (first half vs. second half) for the fraction of invalid samples and trial exclusion rates. 

### 1.7. Participant Characteristics and Data Quality

All 70 enrolled participants completed the three-task protocol. Demographic and clinical characteristics are reported in Table 1. Groups were age-matched (control: 8.7 ± 1.6 years; amblyopia: 9.1 ± 1.7 years) with a similar sex distribution. As expected, the amblyopia group exhibited reduced best-corrected visual acuity in the amblyopic eye and markedly impaired stereoacuity.

Eye-tracking data quality was high across tasks under natural head posture. After blink/off-screen removal and validity screening, the median retained trials per participant were 7/8 for Task 1, 69/72 for Task 2, and 6/6 for Task 3, with no meaningful between-group difference (Table 4). Across retained trials, the mean fraction of invalid samples remained below 8% for all tasks in both groups (Table 4). Based on the retained-trial counts, the corresponding mean trial rejection rates were low overall (approximately 0–11% across tasks) and were similar between groups.

To evaluate potential order-related confounds (Task 1 → Task 2 → Task 3), we compared early vs. late trials within each task (see Table 5). Invalid-sample fractions increased slightly over time in both groups (typically <2 percentage points), and the magnitude of change did not differ meaningfully between groups, suggesting minimal fatigue- or learning-related bias in the data. Together, these results suggest that the fixed task order is unlikely to materially influence the quality-controlled data used for downstream analyses.

### 1.8. Task-Evoked Oculomotor Differences Between Groups

**Figure 3:**
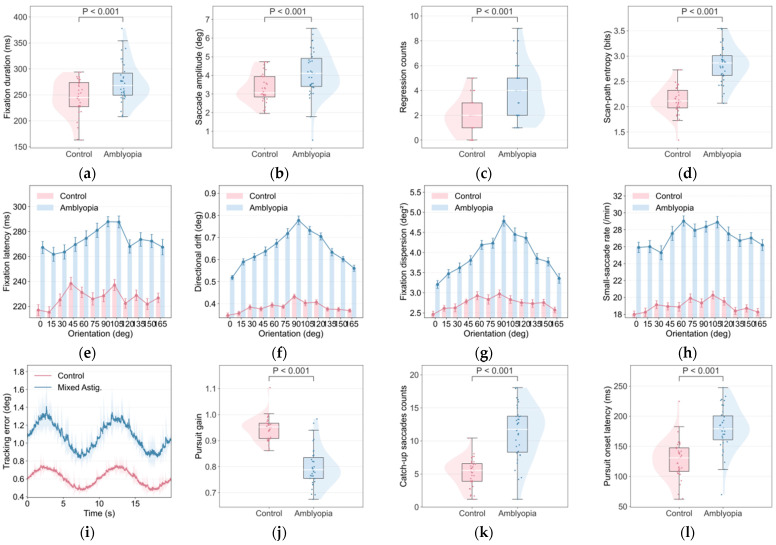
(**a**–**d**) Task-evoked oculomotor differences between control and amblyopic children. (**a**–**d**) Visual enumeration/search: fixation duration, saccade amplitude, regression counts, and scan-path entropy. (**e**–**h**) Orientation-dependent grating viewing: fixation latency, orientation-referenced drift, fixation dispersion, and small corrective saccade-like event rate across grating orientations. (**i**–**l**) Smooth pursuit: tracking error, pursuit gain, catch-up saccades, and pursuit onset latency. Violin/box plots summarize subject-level means; group differences were significant.

Across all three tasks, children with unilateral amblyopia exhibited systematic differences in gaze stability and oculomotor control under best-corrected binocular viewing. Figure 3 summarizes representative feature distributions, and Table 6, Table 7 and Table 8 report subject-level summary statistics and standardized effect sizes. Group comparisons were performed at the subject level using two-sided tests, with false-discovery rate (FDR) control applied within each task’s feature family.

In the visual search/enumeration task (Task 1), the amblyopia group showed less efficient scanning behavior and reduced stability, including higher scan-path entropy, more regressions, and larger inter-fixation step sizes (Table 6; Figure 3a–d; all FDR *p* < 0.001).

In the grating-viewing task (Task 2), amblyopic participants showed delayed stabilization after stimulus onset and increased orientation-referenced drift and dispersion (Table 7; all FDR *p* < 0.001). Orientation-resolved curves further demonstrated larger deviations for oblique orientations (Figure 3e–h), consistent with orientation-dependent processing differences.

In smooth pursuit (Task 3), the amblyopia group exhibited larger position error, reduced pursuit gain, and more frequent catch-up saccades (Table 8; all FDR *p* < 0.001), indicating impaired eye–target coordination under binocular viewing (Figure 3i–l).

### 1.9. Subject-Level Classification Performance and Probability Calibration

**Figure 4:**
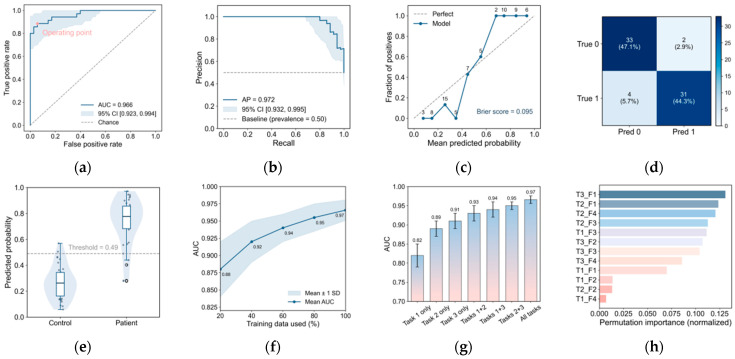
Performance, calibration, and interpretability of the subject-level amblyopia classifier. (**a**) ROC curve for outer-loop held-out subjects; the marked point indicates the inner-loop threshold (maximizing Youden’s J). The shaded region shows the 95% bootstrap band; the dashed diagonal indicates chance. (**b**) Precision–recall curve with 95% bootstrap band; the dashed horizontal line denotes baseline precision (prevalence). (**c**) Calibration plot for aggregated (isotonic-calibrated) subject probabilities (bin-wise observed vs. predicted); the Brier score is reported, and numbers indicate bin sizes. (**d**) Confusion matrix at the selected threshold. (**e**) Distribution of calibrated predicted probabilities by group; the dashed horizontal line indicates the decision threshold. (**f**) Learning curve: mean AUC versus training-set size (%), with the shaded band indicating variability across folds/resamples. (**g**) Task-wise ablation: AUC using each task alone and pairwise combinations, compared with the full model. (**h**) Permutation feature importance (normalized); T1_F1–F4, T2_F1–F4, and T3_F1–F4 denote the four key features from Tasks 1–3, respectively.

Using the full feature set and the nested, subject-wise evaluation protocol, the proposed Random Forest achieved strong discrimination between unilateral amblyopia and controls at the subject level (AUC = 0.966, 95% CI: 0.92–0.99; AP = 0.972, 95% CI: 0.90–0.99; Figure 4a,b; Table 9). Isotonic calibration improved probability reliability for the aggregated subject probabilities (Figure 4c), yielding an expected calibration error of 0.04 and a Brier score of 0.095.

At the operating threshold selected within the inner loop (maximizing Youden’s J), outer-loop held-out predictions yielded TP = 31, FN = 4, TN = 33, and FP = 2 (Figure 4d), corresponding to sensitivity = 0.886 and specificity = 0.943 (Table 9). Aggregated predicted probabilities showed clear separation between groups, with most controls falling below the decision threshold and most amblyopia cases above it (Figure 4e), supporting stable subject-level decisions at the chosen operating point. Learning-curve analysis further indicated that AUC increased with training-set size and showed diminishing returns near the full cohort (Figure 4f).

### 1.10. Baseline Comparisons, Ablations, and Interpretability

To contextualize performance, we trained baseline classifiers on the identical feature set under the same nested, subject-wise protocol. Regularized logistic regression and linear SVM achieved lower AUC and accuracy than the Random Forest, while gradient-boosted trees were comparable but did not exceed the primary model (Table 10).

Task-ablation analyses supported complementary contributions of the three tasks. Single-task models were less accurate than the combined model, and combining tasks improved discrimination, with the full three-task model achieving the highest AUC under the same evaluation protocol (Figure 4g, Table 11).

Model interpretability analyses were consistent with task-level effects. Permutation importance highlighted that the most informative predictors were predominantly derived from the grating-viewing and smooth-pursuit tasks, followed by search-related measures (Figure 4h), suggesting that the classifier leveraged distributed evidence across multiple oculomotor domains rather than a single cue.

### 1.11. Robustness and Sensitivity Analyses

Because recordings were acquired at 30 Hz under natural head posture, we evaluated robustness to reasonable variations in preprocessing and event definitions. Across a grid of fixation-segmentation thresholds (dispersion threshold: 0.75°/1.0°/1.25°; minimum duration: 80/100/120 ms) and two derivative pipelines (Savitzky–Golay vs. low-pass filtering prior to finite-difference velocity), subject-level AUC varied within a narrow range (0.960–0.968) and the top-ranked feature families remained unchanged (Table 12).

We also examined dependence on the modeled gaze stream. Repeating the full pipeline using (i) cyclopean gaze (primary), (ii) amblyopic-eye-only gaze, and (iii) fellow-eye-only gaze demonstrated that the cyclopean and amblyopic-eye-only streams yielded similar performance, whereas the fellow-eye-only stream was lower but remained above chance (Table 13). This pattern suggests that amblyopia-related oculomotor signatures are preserved under binocular viewing, while the amblyopic-eye stream carries the strongest discriminative information.

The clinical-only baseline achieved moderate discrimination, whereas the gaze-based model performed substantially better. Combining clinical variables with gaze features yielded a small additional gain, indicating that gaze dynamics contribute complementary information beyond standard clinical measures (Table 14).

### 1.12. Decision—Analytic Implications Under Real-World Prevalence

Because the study used a balanced case–control design (50/50), positive and negative predictive values will differ in community screening, where prevalence is lower. Using the operating-point sensitivity and specificity estimated above, we computed expected PPV and NPV across plausible prevalence values (Table 15). At 5% prevalence, the expected PPV was approximately 0.45, while NPV exceeded 0.99, indicating that the tool is well suited to rule out amblyopia and to triage children for confirmatory clinical assessment.

## 2. Discussion

This study demonstrates that a brief, task-evoked oculomotor battery yields robust and interpretable behavioral signatures of unilateral amblyopia in school-aged children. Across the three tasks, amblyopic participants showed consistent impairments in gaze stability, saccadic organization, and pursuit fidelity. When these physiologically motivated features were combined in a calibrated Random Forest model, we obtained strong subject-level discrimination and well-calibrated probabilities, supporting the feasibility of task-evoked eye movements as functional screening biomarkers.

Several aspects of the pipeline address common methodological concerns for pediatric wearable eye tracking. All modeling steps were performed within a nested, subject-wise protocol to avoid leakage, with calibration and threshold selection confined to the training data within each split. Data quality remained high across tasks, and although the task order was fixed, order-related changes in the invalid-sample fraction were small and similar across groups, arguing against major fatigue-driven confounding under this short protocol. Importantly, because recordings were acquired at 30 Hz under natural head posture, we evaluated robustness to reasonable variations in preprocessing and event definitions; performance remained stable across these choices. Consistent with this sampling rate and the prototype context, our “small corrective saccade-like event” measure should be interpreted as a coarse index of corrective behavior rather than a canonical microsaccade metric.

Binocular viewing is ecologically appropriate for screening but could in principle attenuate unilateral signatures. We therefore assessed dependence on the modeled gaze stream. Using cyclopean gaze as the primary signal and repeating the pipeline with amblyopic-eye-only and fellow-eye-only signals, we found comparable performance for cyclopean and amblyopic-eye-only inputs, whereas fellow-eye-only performance was lower. This suggests that amblyopia-related oculomotor signatures remain detectable under binocular viewing and that the amblyopic-eye stream carries substantial discriminative information.

The multi-task design also appears to capture complementary information. Task ablation showed that combining tasks improved performance over single-task models, and feature-importance analyses highlighted contributions from pursuit- and grating-derived descriptors, while search-related measures provided additional evidence. For screening applications, such complementarity is valuable because children’s engagement and data quality can vary across tasks and individuals.

With respect to comparators, we benchmarked the proposed approach against standard classical baselines trained under the same nested protocol (regularized logistic regression, linear SVM, and gradient-boosted trees). Comparisons with deep-learning models (e.g., CNNs/transformers) are of interest, but fair evaluation of higher-capacity models typically requires larger datasets to control variance and to support leakage-free hyperparameter selection; thus, deep-learning benchmarking is best pursued in larger multi-center cohorts.

A central translational consideration is that predictive values depend on prevalence. While the present case–control design is balanced, we reported expected PPV/NPV across plausible community prevalence ranges, emphasizing the intended use as a screening/triage tool to refer children for confirmatory clinical assessment rather than as a stand-alone diagnosis. Relatedly, this study did not include a head-to-head comparison against chart-based acuity screening or commercial instrument-based photoscreeners (e.g., Spot Vision Screener) because these modalities were not collected in parallel under matched referral criteria. Prospective validation should therefore include guideline-consistent head-to-head comparisons and external testing on independent cohorts.

## 3. Conclusions

Task-evoked eye tracking combined with calibrated, interpretable machine learning provides a fast and objective approach for screening unilateral amblyopia in children. A brief battery spanning visual search, orientation-dependent viewing, and smooth pursuit revealed consistent oculomotor disturbances that supported accurate subject-level classification and well-calibrated risk estimates under a strictly subject-wise evaluation protocol.

This framework complements conventional acuity-based screening by quantifying functional consequences of amblyopic vision during naturalistic behaviors and can be tuned via threshold selection to support triage in community settings. With prospective external validation, age-specific norms, and streamlined deployment across devices and sites, task-evoked oculomotor assessment has the potential to enable scalable pediatric amblyopia screening and monitoring.

## Figures and Tables

**Table 1:** Demographic and clinical characteristics of participants. Continuous variables are presented as mean ± SD (with range) unless otherwise specified; stereoacuity is presented as median [IQR]. *p*-values reflect between-group comparisons (two-sided): independent-samples *t*-test for variables summarized as mean ± SD, Mann–Whitney U test for stereoacuity, and χ^2^ test for sex. BCVA is reported in logMAR units; spherical equivalent (SE) is in diopters (D). Amblyopia etiology is reported as counts and percentages within the amblyopia group.

Characteristic		Control (*n* = 35)	Unilateral Amblyopia (*n* = 35)	*p*-Value
Age (years), mean ± SD (range)		8.7 ± 1.6 (6–12)	9.1 ± 1.7 (6–12)	0.62
Sex, *n* (male/female)		18/17	19/16	0.81
BCVA (logMAR), amblyopic/worse eye		0.03 ± 0.05	0.34 ± 0.09	<0.001
BCVA (logMAR), fellow/better eye		0.02 ± 0.05	0.05 ± 0.05	0.02
Interocular BCVA difference (logMAR)		0.04 ± 0.03	0.29 ± 0.07	<0.001
Spherical equivalent (D), worse eye		−0.25 ± 0.75	+2.00 ± 2.25	<0.001
Spherical equivalent (D), better eye		−0.15 ± 0.70	+0.50 ± 1.50	0.03
Stereoacuity (arcsec), median [IQR]		60 [40–80]	400 [200–800]	<0.001
Amblyopia etiology, *n* (%)	Anisometropic	—	18 (51%)	—
	Strabismic	—	10 (29%)	—
	Mixed	—	7 (20%)	—

**Table 2:** Task structure and timing.

Task	Trials (Practice + Formal)	Trial Timing	Primary Behavioral Demand
Task 1: Animal icon enumeration	2 + 8	Self-paced; max 60 s	Free visual search and counting
Task 2: Orientation-dependent grating viewing	6 + 72	2 s stimulus + 1 s blank	Attempted central fixation during grating viewing
Task 3: Sinusoidal smooth pursuit	1 + 6	20 s continuous tracking + 3 s pre-fixation	Track a moving target

**Table 3:** Preprocessing and feature-extraction parameters.

Component	Specification
Coordinate units	Pixels converted to degrees of visual angle (60 cm viewing distance)
Cyclopean gaze	Mean of left and right when both valid; otherwise single valid eye; otherwise missing
Invalid sample criteria	Pupil loss/tracking loss; off-screen samples; velocity > 1000°/s; plus ±1-frame margin
Interpolation	Linear interpolation for gaps ≤200 ms (≤6 samples); longer gaps kept missing
Trial exclusion	>30% invalid/missing samples after cleaning
Smoothing	Savitzky–Golay filter (window 5 samples; polynomial order 2)
Velocity estimation	Central finite difference on smoothed positions
Fixation segmentation (I-DT)	Dispersion threshold 1.0°; minimum duration 100 ms
Saccade-like event detection	Velocity thresholding on smoothed signal; amplitude-based classification (see Section 1.5.2)

**Table 4:** Trial retention and sample-level data quality by task (mean ± SD across subjects).

Task	Retained Trials (Ctrl)	Retained Trials (Amb)	Invalid Samples % (Ctrl)	Invalid Samples % (Amb)
Task 1	7.3 ± 0.8	7.1 ± 0.9	6.5 ± 3.2	7.1 ± 3.6
Task 2	69.4 ± 2.1	68.8 ± 2.4	4.2 ± 2.0	4.9 ± 2.3
Task 3	6.0 ± 0.1	5.9 ± 0.3	7.3 ± 3.8	7.8 ± 4.1

**Table 5:** Within-task order-effect check (Early vs. Late; Late-Early). Early and Late refer to the first and second halves (50%/50%) of retained trials within each task for each subject. Δinvalid% was computed as Late-Early. Within-group *p*-values were obtained using two-sided paired Wilcoxon signed-rank tests.

Task	Δinvalid% Late—Early (Ctrl), Mean ± SD	*p* (Ctrl)	Δinvalid% Late—Early (Amb), Mean ± SD	*p* (Amb)
Task 1	+0.4 ± 1.8	0.24	+0.5 ± 2.0	0.19
Task 2	+0.2 ± 1.1	0.41	+0.3 ± 1.2	0.33
Task 3	+0.6 ± 2.1	0.16	+0.7 ± 2.4	0.1

**Table 6:** Group differences for key Task 1 features (subject-level summaries).

Feature	Control (Mean ± SD)	Amblyopia (Mean ± SD)	Median (C/A)	d	FDR *p*
Scan-path entropy (bits)	2.126 ± 0.282	2.824 ± 0.354	2.112/2.865	2.18	<0.001
Regression counts	1.914 ± 1.401	3.857 ± 2.144	2.000/4.000	1.07	<0.001
Saccade amplitude (deg)	3.303 ± 0.776	4.098 ± 1.249	3.066/4.105	0.76	<0.001

**Table 7:** Group differences for key Task 2 features (orientation-averaged; subject-level summaries).

Feature	Control (Mean ± SD)	Amblyopia (Mean ± SD)	Median (C/A)	d	FDR *p*
Fixation latency (ms)	226.5 ± 20.2	272.9 ± 23.5	224.3/273.1	2.25	<0.001
Directional drift (deg)	0.384 ± 0.061	0.646 ± 0.104	0.405/0.624	3.72	<0.001
Fixation dispersion (deg^2^)	2.737 ± 0.312	3.923 ± 0.641	2.760/3.978	2.75	<0.001

**Table 8:** Group differences for key Task 3 features (subject-level summaries).

Feature	Control (Mean ± SD)	Amblyopia (Mean ± SD)	Median (C/A)	d	FDR *p*
Tracking error (deg)	0.605 ± 0.130	1.069 ± 0.210	0.600/1.060	2.16	<0.001
Pursuit gain	0.946 ± 0.046	0.799 ± 0.074	0.952/0.788	2.39	<0.001
Catch-up saccades (count)	5.22 ± 2.07	11.26 ± 4.05	5.53/11.74	1.88	<0.001

**Table 9:** Subject-level performance of the proposed model (nested evaluation).

Metric	Estimate	95% CI
AUC	0.966	0.92–0.99
Average precision (AP)	0.972	0.90–0.99
Sensitivity	0.886 (31/35)	0.74–0.95
Specificity	0.943 (33/35)	0.81–0.98
Accuracy	0.914 (64/70)	0.83–0.96
PPV	0.939 (31/33)	0.80–0.98
NPV	0.892 (33/37)	0.75–0.96

**Table 10:** Baseline model comparisons under identical nested evaluation (subject level).

Model	AUC	Accuracy
Logistic regression (L2)	0.932	0.871
Linear SVM	0.938	0.886
Gradient-boosted trees	0.960	0.900
Random Forest (primary)	0.966	0.914

**Table 11:** Task-level ablation (nested evaluation; subject level).

Feature Set	AUC	Accuracy
Task 1 only	0.862	0.786
Task 2 only	0.931	0.871
Task 3 only	0.910	0.857
Task 1 + Task 2	0.948	0.893
Task 2 + Task 3	0.958	0.900
All tasks (primary)	0.966	0.914

**Table 12:** Robustness of discrimination performance to preprocessing choices (nested evaluation; subject level).

Analysis Factor	Range Tested	AUC Range
Fixation dispersion threshold	0.75–1.25°	0.962–0.968
Fixation minimum duration	80–120 ms	0.961–0.967
Smoothing/velocity estimation	SG(5,2) vs. low-pass	0.960–0.966
Trial exclusion criterion	25–35% invalid	0.961–0.967

**Table 13:** Sensitivity to gaze-stream definition (nested evaluation; subject level).

Gaze Stream	AUC	Accuracy
Cyclopean (primary)	0.966	0.914
Amblyopic eye only	0.961	0.900
Fellow eye only	0.904	0.843
Both-eyes-valid timestamps only	0.962	0.907

**Table 14:** Clinical-only baseline and clinical + gaze combined performance (subject-level LOSO CV; mean [95% CI]).

Model	AUC	Accuracy	Sensitivity	Specificity
Clinical-only (logistic regression)	0.79 [0.69–0.88]	0.73 [0.62–0.83]	0.71 [0.55–0.85]	0.74 [0.58–0.86]
Gaze-only (Random Forest)	0.91 [0.84–0.97]	0.86 [0.76–0.93]	0.87 [0.74–0.96]	0.86 [0.71–0.94]

**Table 15:** Expected PPV/NPV under different prevalence assumptions (sensitivity = 0.886; specificity = 0.943).

Prevalence	Expected PPV	Expected NPV
2%	0.240	0.998
5%	0.449	0.994
10%	0.633	0.987
20%	0.795	0.971

## Data Availability

The data presented in this study are available from the corresponding author on reasonable request. The data are not publicly available due to participant confidentiality requirements.

## Abbreviations

The following abbreviations are used in this manuscript:

AI	Artificial intelligence
AOI	Area of interest
AP	Average precision
AUC	Area under the curve
BCVA	Best-corrected visual acuity
CI	Confidence interval
LCD	Liquid-crystal display
NIR	Near-infrared
ROC	Receiver operating characteristic
ROI	Region of interest
SD	Standard deviation

## References

[1] Hu B., Liu Z., Zhao J., Zeng L., Hao G., Shui D., Mao K. (2022). The global prevalence of amblyopia in children: A systematic review and meta-analysis. Front. Pediatr..

[2] Fu Z., Hong H., Su Z., Lou B., Pan C.-W., Liu H. (2020). Global prevalence of amblyopia and disease burden projections through 2040: A systematic review and meta-analysis. Br. J. Ophthalmol..

[3] Mostafaie A., Ghojazadeh M., Hosseinifard H., Manaflouyan H., Farhadi F., Taheri N., Pashazadeh F. (2020). A systematic review of Amblyopia prevalence among the children of the world. Rom. J. Ophthalmol..

[4] Hensch T.K., Quinlan E.M. (2018). Critical periods in amblyopia. Vis. Neurosci..

[5] Birch E.E. (2013). Amblyopia and binocular vision. Prog. Retin. Eye Res..

[6] Levi D.M., Knill D.C., Bavelier D. (2015). Stereopsis and amblyopia: A mini-review. Vis. Res..

[7] Webber A.L. (2018). The functional impact of amblyopia. Clin. Exp. Optom..

[8] Carlton J., Kaltenthaler E. (2011). Amblyopia and quality of life: A systematic review. Eye.

[9] Grossman D.C., Curry S.J., Owens D.K., Barry M.J., Davidson K.W., Doubeni C.A., Epling J.W., Kemper A.R., Krist A.H., Kurth A.E. (2017). Vision screening in children aged 6 months to 5 years: US preventive services task force recommendation statement. Jama.

[10] Horwood A.M., Griffiths H.J., Carlton J., Mazzone P., Channa A., Nordmann M., Simonsz H.J., Foundation E. (2021). Scope and costs of autorefraction and photoscreening for childhood amblyopia—A systematic narrative review in relation to the EUSCREEN project data. Eye.

[11] Metsing I.T., Hansraj R., Jacobs W. (2018). A review of vision screening methods for children. Afr. Vis. Eye Health.

[12] Sanchez I., Ortiz-Toquero S., Martin R., De Juan V. (2016). Advantages, limitations, and diagnostic accuracy of photoscreeners in early detection of amblyopia: A review. Clin. Ophthalmol..

[13] Arnold R.W., Donahue S.P., Silbert D.I., Longmuir S.Q., Bradford G.E., Peterseim M.M.W., Hutchinson A.K., O’Neil J.W., de Alba Campomanes A.G., Pineles S.L. (2022). AAPOS uniform guidelines for instrument-based pediatric vision screen validation 2021. J. Am. Assoc. Pediatr. Ophthalmol. Strabismus.

[14] Lewis R., Codina C., Griffiths H. (2022). The effect of test method on visual acuity in school children aged 4–5. Br. Ir. Orthopt. J..

[15] Liu Y., Kang M., Gao S., Zhang C., Liu Y., Li S., Qi Y., Nathan A., Xu W., Tang C. (2024). Diagnosis of Multiple Fundus Disorders Amidst a Scarcity of Medical Experts Via Self-supervised Machine Learning. IEEE Internet Things J..

[16] Kelly K.R., Jost R.M., De La Cruz A., Birch E.E. (2015). Amblyopic children read more slowly than controls under natural, binocular reading conditions. J. Am. Assoc. Pediatr. Ophthalmol. Strabismus.

[17] Black A.A., Wood J.M., Hoang S., Thomas E., Webber A.L. (2021). Impact of amblyopia on visual attention and visual search in children. Investig. Ophthalmol. Vis. Sci..

[18] Wang Y., Li S., Chang D., Liu Z., Cheng L., Fu T. (2024). Impaired visual attention and numerical processing in children with anisometropic amblyopia and after visual acuity recovery. Sci. Rep..

[19] Fan Y., Zuo H., Chu P., Wu Q., Li L., Wang Y., Cao W., Zhou Y., Huang L., Li N. (2024). Analyses of eye movement parameters in children with anisometropic amblyopia. BMC Ophthalmol..

[20] Verghese P., McKee S.P., Levi D.M. (2019). Attention deficits in amblyopia. Curr. Opin. Psychol..

[21] Zhao Z., Meng H., Li S., Wang S., Wang J., Gao S. (2025). High-Accuracy Intermittent Strabismus Screening via Wearable Eye-Tracking and AI-Enhanced Ocular Feature Analysis. Biosensors.

[22] Portnoy A., Gilaie-Dotan S. (2025). Oculomotor-related measures are predictive of reading acquisition in first grade early readers. Vision.

[23] Plainis S., Ktistakis E., Tsilimbaris M.K., Gleni A., Simos P. (2025). Implementing silent reading speed and oculomotor behaviour as a clinical measure of functional reading performance. Clin. Exp. Optom..

[24] Niechwiej-Szwedo E., Colpa L., Wong A.M. (2019). Visuomotor behaviour in amblyopia: Deficits and compensatory adaptations. Neural Plast..

[25] Zhu L., Chen J., Yang H., Zhou X., Gao Q., Loureiro R., Gao S., Zhao H. (2024). Wearable near-eye tracking technologies for health: A review. Bioengineering.

[26] Kim T., Lee K.C., Lee K., Baek N., Jung J., Kim E., Park B., Ha J., Kim K.Y., Seo Y.-S. High-speed lensless eye tracker for microsaccade measurement. Proceedings of the SPIE Advanced Biophotonics Conference (SPIE ABC 2023).

[27] Johansson J., Godbolt A., Franzon K., Möller M. (2021). Methodological aspects of using a wearable eye-tracker to support diagnostic clinical evaluation of prolonged disorders of consciousness. J. Rehabil. Med..

[28] Yao B., Zhao Z., Liu Y., Liang Z., Xie E., Gao S. (2025). Automated Strabismus Screening Based on A Wearable Eye Tracker. IEEE Consum. Electron. Mag..

[29] González-Vides L., Hernández-Verdejo J.L., Cañadas-Suárez P. (2023). Eye tracking in optometry: A systematic review. J. Eye Mov. Res..

[30] Spering M., Carrasco M. (2015). Acting without seeing: Eye movements reveal visual processing without awareness. Trends Neurosci..

[31] Hung S.-C., Carrasco M. (2023). Microsaccades as a long-term oculomotor correlate in visual perceptual learning. Psychon. Bull. Rev..

[32] Band T.G., Bar-Or R.Z., Ben-Ami E. (2024). Advancements in eye movement measurement technologies for assessing neurodegenerative diseases. Front. Digit. Health.

[33] Diotaiuti P., Marotta G., Di Siena F., Vitiello S., Di Prinzio F., Rodio A., Di Libero T., Falese L., Mancone S. (2025). Eye Tracking in Parkinson’s Disease: A Review of Oculomotor Markers and Clinical Applications. Brain Sci..

[34] Shaikh A.G., Otero-Millan J., Kumar P., Ghasia F.F. (2016). Abnormal fixational eye movements in amblyopia. PLoS ONE.

[35] Hirota M., Kato K., Fukushima M., Ikeda Y., Hayashi T., Mizota A. (2022). Analysis of smooth pursuit eye movements in a clinical context by tracking the target and eyes. Sci. Rep..

[36] Zhao Z., Li S., Wang J., Li X., Gao S. A Strabismus Widespread Screening Method Based on Wearable Eye Tracker. Proceedings of the 2024 IEEE BioSensors Conference (BioSensors).

[37] Nieboer W., Ghiani A., De Vries R., Brenner E., Mann D.L. (2023). Eye tracking to assess the functional consequences of vision impairment: A systematic review. Optom. Vis. Sci..

[38] Chen D., Otero-Millan J., Kumar P., Shaikh A.G., Ghasia F.F. (2018). Visual search in amblyopia: Abnormal fixational eye movements and suboptimal sampling strategies. Investig. Ophthalmol. Vis. Sci..

[39] Nagarajan K., Luo G., Narasimhan M., Satgunam P. (2022). Children with amblyopia make more saccadic fixations when doing the visual search task. Investig. Ophthalmol. Vis. Sci..

[40] Liao N., Jiang H., Qin Y., Wan M., Zhou J., Lu Z.-L., Yu X., Hou F. (2025). Binocular Contrast Sensitivity Function in Children With Anisometropic Amblyopia. Investig. Ophthalmol. Vis. Sci..

